# Noninvasive Urinary Monitoring of Progression in IgA Nephropathy

**DOI:** 10.3390/ijms20184463

**Published:** 2019-09-10

**Authors:** Joshua Y. C. Yang, Reuben D. Sarwal, Fernando C. Fervenza, Minnie M. Sarwal, Richard A. Lafayette

**Affiliations:** 1 Department of Surgery, University of California San Francisco, San Francisco, CA 94143, USA; 2 KIT Bio, 665 3rd St, Suite 280, San Francisco, CA 94107, USA; 3 Division of Nephrology and Hypertension, Mayo Clinic, Rochester, MN 55905, USA; 4 Division of Nephrology and Hypertension, Stanford University, Stanford, CA 94305, USA

**Keywords:** IgA nephropathy, KIT assay, KIT-IgA score, noninvasive, diagnostics, prediction

## Abstract

Standard methods for detecting and monitoring of IgA nephropathy (IgAN) have conventionally required kidney biopsies or suffer from poor sensitivity and specificity. The Kidney Injury Test (KIT) Assay of urinary biomarkers has previously been shown to distinguish between various kidney pathologies, including chronic kidney disease, nephrolithiasis, and transplant rejection. This validation study uses the KIT Assay to investigate the clinical utility of the non-invasive detection of IgAN and predicting the progression of renal damage over time. The study design benefits from longitudinally collected urine samples from an investigator-initiated, multicenter, prospective study, evaluating the efficacy of corticosteroids versus Rituximab for preventing progressive IgAN. A total of 131 urine samples were processed for this study; 64 urine samples were collected from 34 IgAN patients, and urine samples from 64 demographically matched healthy controls were also collected; multiple urinary biomarkers consisting of cell-free DNA, methylated cell-free DNA, DMAIMO, MAMIMO, total protein, clusterin, creatinine, and CXCL10 were measured by the microwell-based KIT Assay. An IgA risk score (KIT-IgA) was significantly higher in IgAN patients as compared to healthy control (87.76 vs. 14.03, *p* < 0.0001) and performed better than proteinuria in discriminating between the two groups. The KIT Assay biomarkers, measured on a spot random urine sample at study entry could distinguish patients likely to have progressive renal dysfunction a year later. These data support the pursuit of larger prospective studies to evaluate the predictive performance of the KIT-IgA score in both screening for non-invasive diagnosis of IgAN, and for predicting risk of progressive renal disease from IgA and utilizing the KIT score for potentially evaluating the efficacy of IgAN-targeted therapies.

## 1. Introduction

IgA nephropathy (IgAN) remains the most common type of primary chronic glomerulonephritis worldwide [1]. Its prevalence varies among different groups of people, where IgAN makes up about 20–40% of primary glomerular disease in Asia, and about 15–20% in Northern Europe [2]. The onset of IgAN may occur at any age, but the condition most frequently develops in patients in their second and third decades of life [3]. Many patients who present with mild symptoms will not require treatment, though they will be monitored for disease progression, but up to 40% of patients with IgAN will eventually develop end-stage renal disease [1]. Trials have established the benefit of agents that antagonize the renin-angiotensin-aldosterone system (RAAS) in reducing or delaying progression, but even patients treated with RAAS blockade still face deterioration of their kidney function over time [4]. One major issue in the treatment of IgAN is that there are few biomarkers that can predict progression of disease and monitor the efficacy of treatment [5].

Currently, IgAN is diagnosed by renal biopsy which is often performed to confirm causes of mild urinary abnormalities, particularly hematuria; however, biopsies are invasive procedures [6] that carry the risk for complications such as bleeding, and the majority of cases of microscopic hematuria are not associated with any kidney pathology. Recent advances in the analysis of the urinary proteome suggest that the excreted polypeptides include disease-specific patterns [7] and as such, much of the current research has investigated urinary biomarkers of IgA-associated glomerulonephritides. Biomarkers such as urinary neutrophil gelatinase-associated lipocalin or cystatin C [8] and protein/polypeptide patterns [9] have been studied in IgAN; however, their discriminative abilities appear modest and many studies exclude patients undergoing treatment, thus limiting any determination of the monitoring utility of such biomarkers. As a result, there exists a need for a reliable and non-invasive method to diagnose and monitor IgAN, especially in the context of treatment.

Recently, the Kidney Injury Test (KIT) Assay was developed by our group as a noninvasive test to measure kidney injury and function in the context of chronic kidney disease [10], nephrolithiasis [11], and transplant rejection [12]. The KIT Assay measures a panel of urinary biomarkers in a microwell-based format that were identified through a combination of proteomics, genomics, and metabolomics approaches [13,14,15] to be the most sensitive indicators of various etiologies of kidney injury and functional changes. In this investigator-initiated, multicenter, prospective study, we investigated the utility of the KIT Assay, a urine-based, microwell format assay for the detection of patients with IgAN who were treated with standard of care or with Rituximab. We additionally investigated the utility of the KIT-IgA score to predict progression of renal functional decline longitudinally on a subset of these patients who had longitudinally collected samples.

## 2. Results

### 2.1. Study Design and Patient Disposition

**Figure 1:**
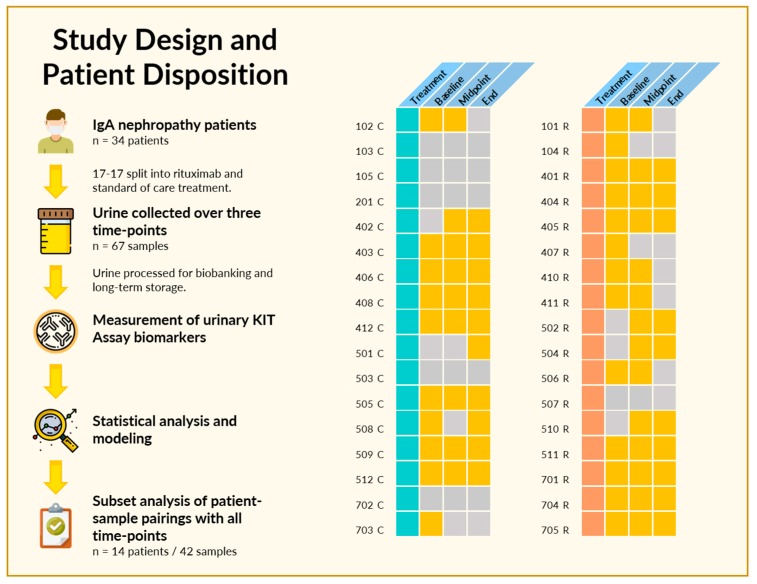
Study design and patient disposition. (Left) In the original trial, 34 patients met inclusion criteria and were randomized into Rituximab and standard of care treatment groups. At least one urine sample was available from 28 of the 34 patients, with 14 having urine samples at all three designated time-points. (Right) Pictorial depiction of patients, treatment, and sample availability. Patients were segregated based on treatment, either with standard of care (turquoise) or Rituximab (coral), with individual patients as rows. A yellow square indicates a urine sample was available at the indicated time-point, while gray indicates that no urine sample was available for analysis due to failure to collect or insufficient sample volume.

Sixty-nine spot urine samples were collected from 34 enrolled patients in the IgAN clinical trial over the study follow-up of 1 year (Figure 1). Patients were randomized at study entry 1:1, to receive either standard of care (corticosteroids) or Rituximab. Patients had biopsy-confirmed IgAN at study entry and serial urine samples were collected at study entry, 6 and 12 months. Two or more samples were available from 25 patients, and a complete set of three time-points were available from 14 patients. The baseline characteristics and disposition of the 34 patients are listed in Table 1.

**Figure 2:**
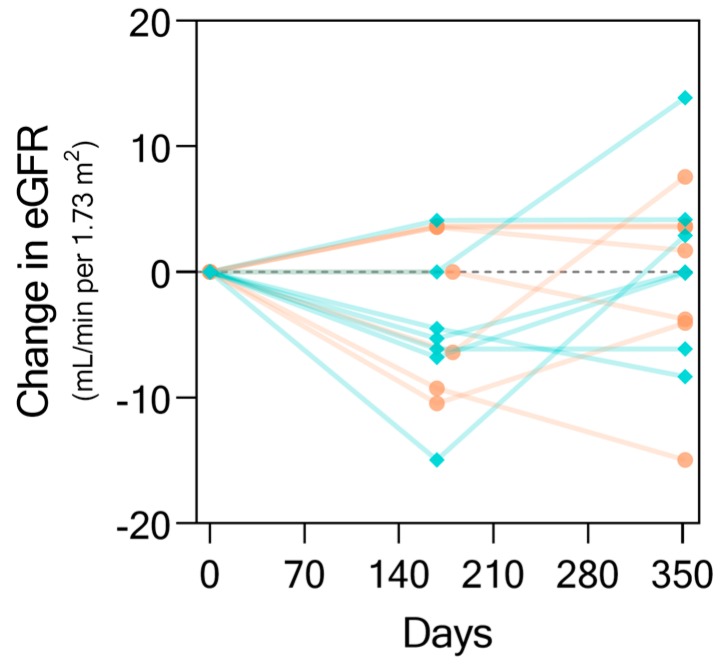
Changes in eGFR (by Modification of Diet in Renal Disease (MDRD) [16]) from baseline. Data shown here for the change in eGFR values over the time-course of the study for a subset of 14 patients who had complete urine samples collected at all three study time-points. Each line represents an individual patient trajectory. Trajectories in teal represent patients who received standard of care while those in salmon represent those who received Rituximab.

As concluded by the original study, and as shown in Figure 2, there was no statistically significant difference between the change in eGFR over the course of the study by treatment with Rituximab (coral) over standard of care (teal). However, while some patients maintained or even recovered kidney function as seen in an increase in eGFR, some patients had IgAN progression with functional decline. This finding motivated us to investigate whether the KIT Assay biomarkers could be used to not only detect IgA nephropathy through urine alone, but also predict and monitor kidney function changes longitudinally.

### 2.2. The KIT Assay Biomarkers Can Discriminate Healthy Controls from Patients with IgA Nephropathy

**Figure 3:**
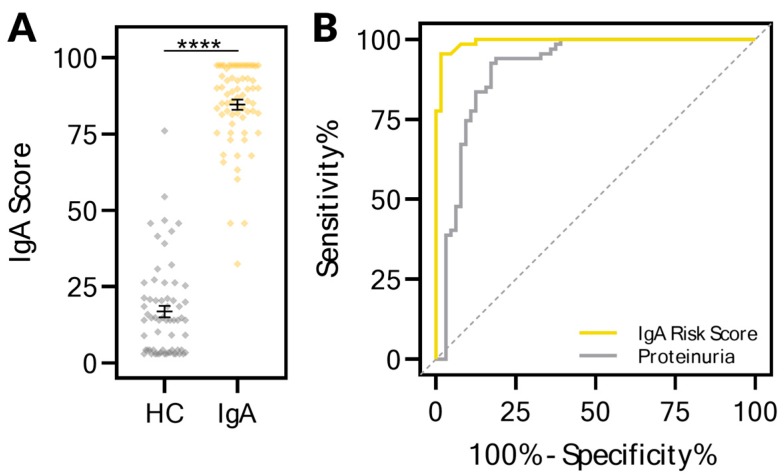
The urinary KIT biomarkers could segregate healthy controls from those with IgA nephropathy. (**A**) An IgA risk score ranging from 0 to 100 segregated healthy control patients from those with IgA nephropathy. Urine samples were collected from healthy controls (*n* = 64) who had no evidence of kidney disease or injury as assessed by both absence of proteinuria and eGFR greater than 120. All urine samples from IgA patients (*n* = 67) were used, as none of these patients had remission of IgA during the treatment duration. (**B**) Receiver–operator characteristic (ROC) curves of the IgA risk score with AUC of 0.994 (*p* < 0.0001) and proteinuria. For the IgA risk score, the sensitivity and specificity were 95.5% and 98.4% respectively. **** *p* < 0.0001.

Urine samples from 64 healthy control patients were assessed and compared to those collected from the IgA nephropathy patients for the KIT biomarkers. A KIT-IgA risk score, ranging from 0 to 100, was developed on these biomarkers using a Bootstrap Forest ensemble model. The KIT-IgA scores for each of the patients in the two groups are depicted in Figure 3A. The KIT-IgA score could distinguish between healthy controls (median 14.03, 95% CI 8.94–18.52) and IgA patients (median 87.76, 95% CI 83.39–90.32) (*p* < 0.0001). Receiver–operator characteristic curves (Figure 3B) comparing the discrimination abilities of the IgA risk score and proteinuria identifies the IgA risk score (AUC 0.9935, 95% CI 0.985–1.000) as performing better than proteinuria (AUC 0.9100, 95% CI 0.855–0.965), suggesting that the KIT-IgA risk score is more sensitive and may detect IgAN earlier than proteinuria. For the KIT-IgA risk score, the sensitivity and specificity were 95.5% and 98.4% respectively.

### 2.3. The KIT Assay Biomarkers Can Discriminate Progressors from Non-Progressors and Predict Progression

**Figure 4:**
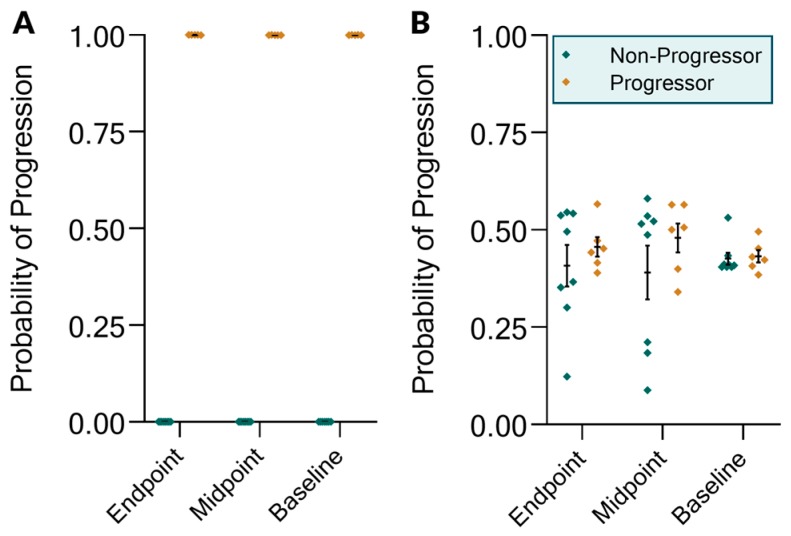
KIT Assay biomarker modeling of progression status after one year of treatment. Modeling was performed on endpoint, midpoint, and baseline biomarker data on either (**A**) the set of KIT Assay biomarkers or (**B**) proteinuria alone. The y-axis shows the probability of progression as determined by a nominal logistic regression model.

We further investigated whether the KIT Assay biomarkers and the KIT-IgA score could distinguish progressors versus non-progressors. Progression was assessed based on a composite clinical evaluation of changes in proteinuria and eGFR from baseline (>50% increase in proteinuria and/or a 25% reduction in eGFR) and, as such, was dependent on both urine and serum biomarker values. We first sought to investigate whether urinary biomarkers alone could be used to classify progressor status. Looking at the 1-year endpoint biomarkers (Figure 4A), progressor status could be classified using nominal logistic regression with 100% accuracy based on urinary measurements alone (*p* = 0.0154). We then investigated whether midpoint (0.5 year prior to progression determination) and baseline (1 year prior) urinary biomarkers could predict progression status. We found that the KIT-IgA score could predict progressor status with 100% accuracy at both time-points (midpoint *p* = 0.0269, baseline *p* = 0.0383). For both the baseline and midpoint predictions, the cfDNA values were the most important predictors, with chi square likelihood ratios of 25.92 and 141.98, respectively, and with *p* < 0.0001 for both. However, neither baseline nor midpoint proteinuria alone could predict progression (Figure 4B). As the progressor definition was based on a composite of proteinuria and eGFR changes, there was no expectation that endpoint proteinuria alone could predict progressor status, as reflected in the probabilities. We further assessed the ability of the baseline urinary biomarkers to predict progression status on the entire set of patients who had baseline urine samples, regardless of the presence of additional longitudinal samples. For these 23 urine-patient pairs, the results were similar to the original analysis in that the KIT biomarkers could segregate progressors from non-progressors with 100% accuracy (*p* < 0.0001) (ROC AUC = 1.00).

## 3. Materials and Methods

### 3.1. Patients and Study Characteristics

Samples were those from a study done in 2017 (NCT00498368) and the study was registered with clinicaltrials.gov [17]. Patient inclusion and exclusion criteria were as described in the previous study. Subjects included adults, ages 18–70 years old, with biopsy-proven IgAN within 2 years of enrollment were included. Patients were excluded if their biopsy showed >50% glomerular sclerosis or interstitial fibrosis or >10% glomerular crescents. Subjects were then randomly assigned to receive Rituximab or continue standard care. Baseline Oxford classification score [18] was recorded in blinded fashion by an expert renal pathologist. eGFR (by Modification of Diet in Renal Disease (MDRD) [16]) or measured creatinine clearance had to be <90 and >30 mL/min per 1.73 m^2^. To establish continued risk, baseline proteinuria needed to be >1000 mg/day while on stable doses of angiotensin-converting enzyme inhibitor, angiotensin receptor blocker, or renin inhibitor therapy for at least 2 months. However, patients who were on dual therapy with agents that inhibit angiotensin II required a lower proteinuria threshold of >500 mg/day. Baseline BP was controlled to <130/80 mmHg. Patients with secondary forms of IgAN, such as cirrhosis, were excluded, although subjects with Henoch–Schönlein purpura nephritis (HSPN) could be included. Patients were excluded if they had previously received Rituximab, were receiving other immunosuppressive therapy, or had ever received >6 months of prednisone or other systemic corticosteroid therapy in the past. There was no corticosteroid exposure within 3 months of study initiation. Randomization was done centrally by a random assignment by prefilled envelopes. The protocol was approved by the institutional review board of each participating center. The study was registered with clinicaltrials.gov, protocol NCT00498368. Informed consent was obtained before all study procedures, and the study adhered to the Declaration of Helsinki.

The primary outcome measures were the change in proteinuria and the change in eGFR from baseline to 12 months. The secondary outcome was safety related, comparing overall adverse events and monitoring for potential intervention-specific complications, such as infusion-related reactions, hypogammaglobulinemia, and infections. Progression was defined as a >50% increase in proteinuria and/or a 25% reduction in eGFR at the one-year time-point. Results showed that treatment with Rituximab resulted in statistically insignificant changes in proteinuria or partial remissions that could not be distinguished from the response to supportive therapy alone.

For the healthy control cohort, urine samples were collected from healthy controls (*n* = 64) who had no evidence of kidney disease or injury as assessed by both absence of proteinuria and eGFR greater than 120. Demographics of this cohort include a median age of 24 (range 2 to 64), median weight of 57.5 kg (range 13.7 to 120.2), 53.1% female, 9.4% African American, and median eGFR of 132 (range 120 to 213).

### 3.2. Urine Sample Processing

Voided urine samples were collected in sterile containers. Urine samples were centrifuged at 2000× *g* for 30 min at 4 °C. The supernatant was separated from the urine pellet containing cells and cell debris. The pH of the supernatant was adjusted to 7.0 using Tris-HCl and stored at −80 °C in the UCSF Biorepository until further analysis.

### 3.3. KIT Assay Biomarkers

Measurement of the KIT Assay biomarkers was performed as previously described [10]. Total protein was measured using the Pierce^TM^ Coomassie Plus Assay Kit (Thermo Fisher Scientific, Waltham, MA, USA). Urinary creatinine was measured using the Creatinine Assay Kit (BioAssay Systems, Hayward, CA, USA) as a method for urinary concentration and hydration status normalization. Briefly, cell-free DNA (cfDNA), methylated cell-free DNA (m-cfDNA), CXCL10, Clusterin, (Dimethylamino)iminomethyl-ornithine (DMAIMO), and (Methylamino)(methylimino)methyl-ornithine (MAMIMO), were measured using the KIT Assay (KIT Bio, San Francisco, CA, USA), a set of in-house developed ELISA assays. Microwell plate readings were measured using a SpectraMax iD3 Multi-Mode Microplate Reader (Molecular Devices, San Jose, CA, USA). All assays were run in duplicates.

### 3.4. Statistical Analysis

Comparisons involving two groups were performed using the nonparametric Mann–Whitney *U* test. Comparisons involving three groups were performed using the nonparametric Kruskal–Wallis test with Dunn’s post-hoc multiple comparisons correction. The IgA risk score was determined using a Bootstrap Forest ensemble model by averaging numerous decision trees each fit to a bootstrap sample of the training data. The final prediction is the average of the predicted values over all the decision trees. Specifically, six trees were used in the forest, with a minimum of ten splits per tree and a minimum size split of five. The number of splits for trees 1–6 were 9, 17, 15, 7, 13, and 11 respectively. The top contributing biomarkers as based on G^2^ were total protein, MAMIMO, creatinine, and DMAIMO, with G^2^ values of 36.02, 21.12, 9.72, and 6.74 respectively. The overall statistics for the Entropy R^2^ was 0.7422, Generalized R^2^ was 0.8568, and Root Mean Square Error (RMSE) was 0.1996.

Modeling of progressor status was done using nominal logistic regression. All analyses were performed using either GraphPad Prism 8.0.2 (GraphPad Software, Carlsbad, CA, USA) or JMP 14.3 (SAS Institute, Cary, NC, USA).

## 4. Discussion

In this study, we used urine samples from IgA nephropathy (IgAN) patients to evaluate the predictive capacity of the KIT Assay biomarkers to discriminate IgAN from healthy controls as well as predict progression of renal functional decline. Our results show that these urinary biomarkers could distinguish between healthy control patients and those with IgAN better than urinary protein alone. Furthermore, the biomarkers could predict progressor status up to one year in advance of status determination while urinary protein could not.

Such findings are important because IgAN is the most prevalent chronic glomerular disease worldwide [19], with diagnosis historically requiring kidney biopsy. While recent studies have identified serum and urinary biomarkers that can diagnose IgAN with high accuracy [7,8,20], the clinical utility of individual biomarkers is limited by the high overlap between the healthy and diseased ranges of these biomarkers in an individual and temporal manner. Such biomarkers, while indicative of IgAN, have shown less utility in predicting progression of disease. While some studies have suggested the use of GC–MS and similar technologies to identify a panel of biomarkers, these proteomic studies are not easily transferrable to the clinical setting. Identifying biomarkers that not only can identify IgAN but can also predict progression is important for the future development and evaluation of therapies that seek to treat and prevent functional decline in IgAN.

The multi-hit hypothesis for the pathogenesis of IgA nephropathy is the leading theory behind the mechanism of progression [20]. In this hypothesis, deposition of IgA1-containing circulating immune complexes in the glomeruli induce renal injury by subsequent induction of local cytokine, growth factor, and complement system production and activation [21,22,23,24]. While currently approved therapies for IgAN are largely non-IgAN specific and consist of supportive therapy with corticosteroids and blood pressure control [25], future directed therapies targeting the specific “hits” in IgAN will address these acute causes of glomerular injury. Monitoring the efficacy of such therapies will require biomarkers that not only reflect kidney function, but also convey the acute status of kidney injury and resolution of that injury.

We had previously validated the utility of the biomarkers comprising the KIT Assay in chronic kidney disease [10], chronic lung transplant rejection [26,27], nephrolithiasis [11], and kidney transplant rejection [12,28], suggesting its utility across a range of diseases and chronicity states. The KIT Assay biomarkers are measured in a microwell format, contributing to their ease of use and deployment. Additionally, urine is a completely noninvasive biofluid that is available in virtually unlimited quantities, enabling a frequency of monitoring that can be adjusted to the functional status of the patient. Due to the acute and chronic injury mechanism present in IgAN [29], we investigated whether the KIT Assay biomarkers could also be applied to detection of IgAN as well as prediction of progression status.

We have discussed the rationale of the KIT Assay biomarkers previously [10], with selection of these biomarkers due to the various temporal and regional differences in levels as dependent upon renal injury and function. While proteinuria has been shown to be a predictor of outcome in IgAN [30] and is a commonly used endpoint, proteinuria did not perform as well as the composite IgA risk score in discriminating patients with IgAN from healthy controls, likely because many patients with IgAN present with low levels of proteinuria despite disease progression [31]. That the KIT-IgA risk score could identify IgAN more accurately is likely due to the composite integration of functional, injury, and renal-specific markers that may reflect IgAN across a spectrum of disease states.

Further, proteinuria alone was not a significant predictor for progression at one-year of treatment duration measured at either the baseline or midpoint time-points. It is notable that the cfDNA values at baseline and midpoint were the strongest predictors of progression at the one-year timepoint. Urinary cfDNA has been previously reported to be a sensitive indicator of kidney injury and reflects ongoing damage to the kidney prior to functional changes or decline [15,32,33]. This finding is in concordance with these reports, suggesting that while progression may be defined by functional measures such as eGFR and proteinuria, the underlying cause of such progression may be acute injury such as that measured by cfDNA burden. As such, measurement of cfDNA, as done in the KIT Assay, may be beneficial for measuring the efficacy of treatment in IgAN.

In summary, our data indicate that the non-invasive KIT Assay panel of urinary biomarkers can distinguish between healthy controls and patients with IgAN. Furthermore, this panel could monitor progression of disease and changes in kidney function. We believe that the KIT-IgA score would have great utility in (1) screening for IgAN in patients with hematuria, as a replacement for biopsy as done in many Asian countries; (2) diagnosis; (3) monitoring efficacy of treatment; and (4) predicting progression and monitoring disease activity. While our sample size is limited by the small size of the parent study, these results add to a growing body of evidence of the generalizability and utility of the KIT Assay biomarkers in renal disease and beyond. Additionally, while all comparisons in this study were between healthy control and IgAN patients, we have planned additional studies where we will include samples from patients with other types of renal diseases so that we can assess the performance of these and other biomarkers in discriminating IgAN from other nephropathies. Future studies using additional kidney disease cohorts will clarify whether there is a specific pattern among these biomarkers to discriminate one disease from another. Future multi-center, prospective studies are planned to further build on the clinical utility of these observations to assess serial performance of the KIT assay in IgAN patients as a non-invasive tool to monitor disease progression and evaluate the efficacy of new reno-protective therapies for IgA disease.

## Figures and Tables

**Table 1:** Baseline characteristics of IgA nephropathy (IgAN) patients.

Baseline Characteristics	IgA Cohort (*n* = 34) ^1^
Age, years	40 (21–63)
Sex	
● Female	9
● Male	25
Race	
● Caucasian	24
● Asian/Pacific Islander	6
● Hispanic/Latino	3
● African American	1
Weight, kg	89.7 (57–120)
eGFR, mL/min per 1.73 m^2^	49 (30–122)
Treatment	
● Rituximab	17
● Standard of Care	17

^1^ Data are reported as median (range) or count. *n* indicates the number of patients in the cohort.

## Abbreviations

AUC	area-under-curve
BMI	body mass index
cfDNA	cell-free deoxyribonucleic acid
DMAIMO	(dimethylamino)iminomethyl-ornithine
eGFR	estimated glomerular filtration rate
GC–MS	gas chromatography–mass spectrometry
HSPN	Henoch–Schönlein purpura nephritis
IgAN	immunoglobulin alpha nephropathy
KIT	Kidney Injury Test
m-cfDNA	methylated cfDNA
MAMIMO	(methylamino)(methylimino)methyl-ornithine
MDRD	Modification of Diet in Renal Disease
RAAS	renin–angiotensin–aldosterone system
ROC	Receiver-operator characteristic
